# Neither the Third Nor the Fourth Age: Viewing Old Age Through the Philosophical Lens of Ambiguity

**DOI:** 10.1093/geront/gnad113

**Published:** 2023-08-26

**Authors:** Susan Pickard

**Affiliations:** Department of Sociology, Social Policy and Criminology, University of Liverpool, Liverpool, Merseyside, UK

**Keywords:** Frailty, Merleau-Ponty, Paradox, Simone de Beauvoir, Successful aging

## Abstract

This paper argues that the philosophy of ambiguity, associated with Maurice Merleau-Ponty and Simone de Beauvoir, provides a helpful framework for understanding the complex and paradoxical nature of old age outside the dominant categories of the “third” and “fourth” ages. Building on philosophical literature in cultural gerontology including that focused on the “art of living” and other approaches that draw on existentialist thought, it suggests “ambiguity” as a theme that has been overlooked in such literature. The advantage of this approach is that it can accommodate contradictory elements and thus underpin an integrated approach to old age. At the same time, in distinguishing between ontological and social dimensions of ambiguity, the conceptual framework offers a new perspective on ageism that can help clarify the distinction between ageism as oppression and the negative aspects of deep old age itself. The paper is structured as follows. After making the case for the value and importance of “ambiguity” as a framework for viewing old age, I set out the key aspects of this philosophy as found in the work of Merleau-Ponty and particularly Beauvoir. I then apply this framework to a discussion of 2 literary memoirs of deep old age, by Diana Athill and Florida Scott-Maxwell, respectively. I argue that the picture of old age that emerges contrasts with the hegemonic discourses enframed by successful aging and frailed old age and offers the possibility not only of a true appreciation of old age but also of a more meaningful life course itself.

## Background and Introduction

Many scholars have called for a more integrated approach toward the reality of aging, bringing together the “positive” aspects of old age with its “shadow,” or those aspects of deep old age that are associated with the “fourth” age. While, in lived experience, positive and negative aspects of aging are threaded together, there are consequences arising from retaining this split on the social, cultural, and individual levels, which have ramifications well beyond old age itself. Many of these have been discussed extensively in the literature, including the association between successful aging and youthfulness in both corporeal and psychosocial dimensions, the resulting stigma associated with the fourth age, and the more general hollowing out of meaning from the life course. More recently, Higgs and Gilleard (2020, 2021, 2022) have suggested a further consequence, which is that of age scholars using the concept of ageism to refer to any negative view about, or attributes observed in, old age. This, they suggest, renders ageism too diffuse and multifaceted to distinguish clearly between what is a constructed negativity on the part of a society and culture intent on devaluing old age and what has its origin in unwelcome changes, vulnerabilities, and challenges especially in terms of the corporeal.

The imperative to keep the elements of good and bad aging apart has been intensified in late modernity through the emergence of the neoliberal model of the life course. In associating good old age with youthfulness and bad old age with senescence, this split has deprived old age of any intrinsic or unique value and meaning (Cole, 1992). Here I should clarify that although neoliberalism and (late) modernity are certainly not synonymous, there is a direct lineage between enlightenment thinking and neoliberalism. As Clack and Paule sum up: “If the thinkers of the European enlightenment defined the self as rational, autonomous and capable of choice, the last forty years have seen these ‘choices’ shaped through the activities of consumer capitalism” (2019, p. 3). As Rose (1999) has further explicated, in neoliberalism, individuals’ “free” choices are aligned with the aims of government shaped through both the market and expert discourses and realized through new forms of consumption, identities, and lifestyle valorized at each stage of the life course. In the case of old age, the neoliberal discourse counterposes successful aging, with its emphasis on individual health underpinned by an active and socially engaged lifestyle, to frail old age, or the fourth age (Rubinstein and de Medeiros, 2015; Shimoni, 2018). This admits to no sense of growth or change through the life course: successful aging, temporally speaking, is resistance to change from the youthful ideal. Similarly, the fourth age has no connection with what preceded it but is depicted as a stage wholly disconnected from the others. Within such a philosophical and cultural context, the pleasures, gains, and possibilities of old age as the final stage of a whole life course become inconceivable. Indeed, in recent years, wealthy Silicon Valley entrepreneurs have made conquering aging (and death) the ultimate status symbol (Appleyard, 2008). An alternative framework(s) for meaning is thus important, which, as Cole suggests, “will require a new and integrated appreciation of ageing that transcends our historical tendency to split old age into positive and negative poles” (1989, p. 381). It will also require powerful alternative concepts and discourses from those that underpin the neoliberal life course, including alternative approaches to freedom, choice, and agency as well as understandings of well-being and satisfaction that lie outside the dimensions of success and failure.

There are powerful philosophical approaches on which we might draw for this project. Some of these, moreover, portray a more complex, paradoxical, and intricate existential reality, a chiaroscuro of light and shade. One such is the philosophy of ambiguity, which offers an interesting and powerful alternative to the neoliberal model of the life course and its approach to old age and aging. In suggesting ambiguity as an appreciative and sensitive framework, this paper aims to make a small contribution to the insightful philosophical literature in the gerontological field focused on the “art of living” (e.g., Baars, 2017; Cole, 1992; Dohmen, 2013; Laceulle 2018; Moody, 1986; Tornstam, 1997). This literature shares an emphasis on the possibilities of old age in terms of growth and self-realization through recognition of vulnerability and finitude. Seeking to provide an alternative, as Dohmen puts it “to the dominant neoliberal concept of the choice biography” (2013, p. 31), this body of work suggests that old age can and should be seen both as a unique stage of life and at the same time part of a more coherent and meaningful life course. However, although often drawing on existentialist thought, ambiguity has been overlooked by this literature and its value as a philosophical principle that can underpin an alternative view of old age remains unexplored.

The philosophy of ambiguity is associated particularly with Simone de Beauvoir building on the foundational work of Merleau-Ponty and when applied to later life, it proposes a different way of representing old age including the good, the bad and the everyday aspects. It also permits a finely graded distinction to be made between the ontological condition of old age situated within a finite life course, which necessarily comprises vulnerabilities as well as strengths, gains as well as losses, and the social limitations and negative judgments imposed by ageism. Moreover, ambiguity rejects modernity’s quest for clear categorizations and classifications, the “either/or,” insisting instead on the ontological primacy of paradox and the presence of “and/but” as ever-present in lived experience. In this way it can serve as an important counterpoint to the hegemonic discourses of contemporary society.

In the next section of the paper, I will set out some of the key elements of the philosophy of ambiguity. As well as exploring how ambiguity replaces order and the neat, yet distorting, polarities and categorizations through which modernity functions with an altogether more paradoxical universe, I will also explicate how this theory conceptualizes ageism. In comparison with Merleau-Ponty’s work, Beauvoir possessed a somewhat more sociological focus, concerned with the way certain groups in particular societies are denied freedom, either by closing down ambiguity or by imposing upon them more, and problematic, forms of ambiguity arising from structures of power. This theory allows us to understand ageism (and sexism and racism) as separate to, though intertwined with, the existential conditions of old age, therefore facilitating the precision advocated by Higgs and Gilleard. I discuss the theory in more depth next.

## Ambiguity: Existential and Social

As Tidd describes, ambiguity as a concept “resists easy definition”; it consists of the contradictions and paradoxes that inhere in the fact that we are simultaneously “both free and unfree, separate and connected to each other, a subject for ourselves and an object for others, consciousness and body, alive yet born to die” (Tidd, 2004, p. 38). This perspective directly challenges Cartesian dualism, the philosophy underpinning modernity, which Beauvoir saw as setting out to deny the inherently paradoxical nature of existence. She writes of these dualist philosophers: “The ethics which they have proposed to their disciples has always pursued the same goal. It has been a matter of eliminating the ambiguity by making oneself pure externality, by escaping from the sensible world or by being engulfed by it” (1976, p. 6). Ambiguity is antithetical to the Cartesian project, which aims to achieve certainty and argues “whatever I clearly and distinctly perceive … is necessarily true” (Descartes, 1970, p. 63; quoted in Langer, 2003). By contrast, ambiguity emphasizes “a commitment to ontological pluralism and freedom, to the dependence of reason on experience and to philosophy being descriptive of experience rather than an escape from experience” (Sapontzis, 1978, p. 542). As a result, the philosophy of ambiguity utilizes the methods of description and particularly phenomenological description (including through fiction and memoir), which operates not through abstract reasoning or objective claims but by setting out concrete experience in all its rich paradox without trying to explain or resolve it. To Merleau-Ponty, the essential ambiguity of human existence results from several factors, in particular the nature of human embodiment and the nature of perception, temporality, and experience that arises from this (Silverman, 1979). Through embodied being one is both subject and object, without even being reducible to one (pure consciousness) or the other (pure facticity). One famous example that Merleau-Ponty gives is that of one hand touching the other. In *The Phenomenology of Perception* he writes: “When I press my two hands together, it is not a matter of two sensations felt together as one perceives two objects placed side by side, but an ambiguous set-up in which both hands can alternate the roles of touching and being touched” (Merleau-Ponty, 2008, p. 106). As Sonia Kruks comments, “It implied that the philosopher could not conceive of himself as a detached ‘spectator’ of reality but was always a situated participant” (2019, p. 116). The reference to touch also suggests an alternative to the dominance of the visual, which is associated with this spectator theory of knowledge. By contrast to this, Merleau-Ponty posits a “corporeal consciousness” (Langer, 2003, p. 102) in which the self and world relate through a looping dialectic: “The bodily senses are themselves inseparably intertwined, forming an ‘intentional arc’ that projects an anticipated world” (Langer, 2003, p. 103). Time and space are also part of this and for Merleau-Ponty, space and time are inseparable from one’s subjectivity: “My body appears to me as an attitude directed towards a certain existing or possible task. And indeed its spatiality is not … a *spatiality* of *position,* but a spatiality of *situation*” (2008, pp. 114–115). Similarly, in terms of lived temporal experience, one’s past, present, and future all coexist. Merleau-Ponty writes, of consciousness, that “at every moment, its former experience is present to it in the form of a horizon which it can reopen … in an act of recollection, but which it can equally leave on the fringe of experience, and which then immediately provides the perceived with a present atmosphere of significance” (p. 25). For Beauvoir, as for Merleau-Ponty, time cannot be extricated from lived experience. Referring to the contemporary idea of “saving” time by technology or efficiency, Beauvoir notes that for lived time: “it is contradictory to want to save up existence, which, the fact is, exists only by being spent” (1976, p. 85). She observes that the present “is the moment of choice and action; we cannot avoid living it through a project” (Merleau-Ponty, 2008, p. 82) and “it is not a question of stopping the movement of life: it is a question of fulfilling it” (p. 86). This gives a rather different perspective to the place of time in old age compared to other stages, as well as to the experience of moving through the life course.

he embodied self is situated, then: in time, space, culture, biology, and discourse. Beauvoir is particularly interested in the implications of this for women (in *The Second Sex*) and older people especially (in *The Coming of Age*) and in exploring how the “situation” one occupies impacts on freedom and, at worst, contributes to oppression. While in everyday life, individuals fluctuate between being both a subject (for themselves) and an object (for others), for dominant groups, the object position is only fleeting, whereas others find themselves fixed more enduringly in this position (through the objectification generated by the male gaze and the youthful structure of the look, for instance). Because “freedom” as a concept is central to both neoliberalism and the existentialism of Beauvoir, it is worth delineating the difference in the meaning attributed to freedom in each system. As Rose observes, freedom is central to neoliberalism and “the associated celebration of the powers of the individual, of autonomy and choice, underpin attempts to specify and construct new forms of social arrangements” (1999, p. 64). Neoliberalism as a mode of governance depends upon the capacities of free individuals but is, above all, a “formula of power” (p. 65) consistent with welfare retrenchment, privatization of pensions, and a free market. Ambiguity, however, suggests an ontological freedom—the human condition as one of constant becoming without predetermination. This means that my situation and the greater or lesser freedom resulting from it for me never fully determine my response to the world. In fact, through a process of reflection and questioning, I allow a distance to open up between myself and my situation that makes possible authentic choice, in however great or limited a dimension. Freedom here contrasts with the agency implied in the neoliberal emphasis on choice as action; it refers to this distance or space of possibility required to make choices and craft thereby our own meaning (Wilkerson, 2017). Without such freedom, ethics is not possible. Again, there is an emphasis on the individual in both neoliberalism and the philosophy of ambiguity but unlike in neoliberalism where old collective solidarities and class identities are replaced by an individualized society, in the philosophy of ambiguity, “it accords to the individual an absolute value” but at the same time “the individual is defined only by his relationship to the world and to other individuals … and his freedom can be achieved only through the freedom of others” (Beauvoir, 1976, p. 169).

Another important element of our ontological ambiguity is our finitude, the paradox being that, although “every living movement is a sliding toward death” yet also “every movement toward death is life … the present must die so that it may live” (1976, p. 137). Finitude, in this framework, is not a property of the old but something that gives meaning to the entire life course. Without finitude, as Beauvoir shows in her achingly evocative though lesser-known novel, *All Men are Mortal*, there would be no value or meaning in the experiences of life. Count Fosca, the immortal protagonist of this novel, expressing the melancholia of his situation in powerful yet simple terms, remarks: “I looked hard at the saffron-coloured rose but there had been too many roses in my life, too many springtimes” (2003, p. 301) for him to distinguish one from another, and care.

Seen through the lens of ambiguity, oppression, including when it takes the form of ageism, arises through the closing down of possibilities otherwise kept open, particularly through “fixing” people or groups in the “object” position and obstructing them from fulfilling their projects and transcending the given situation. Alongside this it takes the form of a troubling form of ambiguity in which older people’s subjectivities are doubled, becoming simultaneously subjects and objects for themselves. As Beauvoir puts it: “Old age is a dialectic relationship between my being as he defines it objectively and the awareness of myself that I acquire by means of him. Within me it is the Other—that is to say the person I am for the outsider—who is old: and that Other is myself” (1996, p. 284). For women as they age, there is a further gendered dimension in that the objectification they have experienced all their lives through the fact of being women in a patriarchal regime can lead to a more negative but equally objectifying experience of devalued femininity at midlife and later, through the double standard of aging (Pickard, 2022).

This “othering” serves an important psychological role for the dominant groups in allowing them to evade the uncomfortable paradoxes of existence. Julia Twigg has observed that while the feminist gerontological literature has often written “from the inside” of the third age, the same is not true of the fourth age. Depictions of the latter are “distant, objective, often couched in officialese or the language of policy-makers. Written from the outside, it is about *them*—the old—not us” (2004, p. 64). As a result, Twigg continues: “These old remain eternally Other; and that sense of them as a wholly separate and as a fundamentally different category of being lies at the heart of how ageism operates” (p. 64). Gilleard and Higgs have suggested that this distancing takes place via the construction of the fourth age as a category that totally separates those individuals perceived to have entered it. In a metaphor that has become very influential, they suggest that for these older people it is as if they have moved over the rim of an event horizon into a black hole: “Any light emitted from beyond this horizon can never reach the observer,” they write (2010, p. 125). No longer individuals, they are frightening archetypes of senescence. Those few empirical studies and literary accounts that do privilege the experience of individuals in advanced old age, however, show us individuals who discover, in challenges, opportunities to grow toward valued ends; or enjoy the present without either fearing the future or feeling disconnected from their pasts (Tornstam, 1997). This does not mean that older people are easily able to discard neoliberal scripts or to leave behind norms and expectations that were relevant to earlier stages of the life course; on the other hand, retaining the values of youth can bring a sense of loss and disappointment to old age (Tornstam, 1997). Dohmen (2013) suggests that if choice is reimagined not within the terms of neoliberalism but rather with respect to authenticity, lifestyle then becomes a moral framework through which to experience old age. This will require a dual process of discarding values that now no longer serve one well and introducing new goals and values. The emphasis within this process, moreover, is on life in terms of an “open future,” recognizing that one can continue to grow in deep old age and right up to and including when one is dying, as the philosophy of ambiguity suggests.

In the next section, and following Twigg’s point about the harmful scarcity of “insider” accounts of this life stage, I will explore two literary memoirs that discuss deep or advanced old age in such a way as to enable the reader to connect imaginatively with the existential situation of that life stage. Contemplating this rich phenomenological material through the prism of ambiguity helps us to view the paradoxical nature of the existential situation in each case as elements of a whole, like the facets of a diamond, rather than attempting to divide, separate, or classify in the usual ways.

## Deep Old Age as Ambiguous: The View From the Inside

### Diana Athill

A former literary editor, born in 1917 in England, one journalist wrote of her that after a life of moderate success, her “rocket really shot into the sky” (Preston, 2011) at the age of 90 with the publication of her first memoir of old age. Both Athill’s memoirs of deep old age (2008, 2015) convey its ambiguity in boldly vivid terms and demonstrate old age to be a stage with its own norms. While a deeply personal account, rich with its own particularity, it also gives the (younger) reader a more general insight into the spatial and temporal situation of this life stage and creates a bridge from her embodied situation to those of other younger readers. The opening notes of her first memoir of old age (2008) are imbued with sadness as she opens with two feelings of loss. The first is the reflection that she can never again own a puppy as she would never be able to walk it. The second is when, ordering a tree fern through a catalogue, it arrives as a seedling: she is certain she will not live to see it grow large in the garden and thrive. Although the note of melancholy never fades entirely, however, Athill quickly goes on to make clear that there are gains aplenty to harvest in this time of life. For example, through taking up drawing in old age, she feels phenomenologically that her senses are expanding in old age, not contracting. As a result of those classes taken in her 70s and 80s “I am now much better at seeing things than I used to be … even in old age … it adds such a generous pinch of pleasure to one’s days” (p. 94). She also discovered later in life that she had a talent for writing when she published her first novel. Paradoxically, however, this was accompanied by the realization that:

none of it mattered at the deepest level, so that all of it could be taken lightly. When you are young, a great deal of what you are is created by how you are seen by others, and this often continues to be true even into middle age … In my eighties that couldn’t happen, no event could be crucial to my self-esteem in quite that way any more, and that was strangely liberating (p. 155).

The melancholy tone remains but it is mixed with contentment as she reflects: “It meant some sort of loss, I suppose, such as the end of thrilling possibilities; but it allowed experiences to be enjoyable in an uncomplicated way—to be simply fun” (p. 155).

There are also strongly gendered dimensions to Athill’s account, which portray femininity in deep old age as an interlaced pattern of loss and gain. Firstly, Athill recognizes her own, greater, freedom manifested in her choice of vibrant outfits in comparison to the restrictive dress codes of her grandmothers’ generation, which emphasized the need for older women’s invisibility and as a result, she writes: “I know for sure that I both feel and behave younger than my grandmothers did when they were old” (p. 15). Then there is her sexuality and here there is a putting away of things that belong to youth. Moving into her 70s, she reflects, signaled the disappearance of what used to be the most important thing in her life:

I might not look, or even feel, all that old, but I had ceased to be a sexual being, a condition which had gone through several stages and had not always been a happy one, but which had always seemed central to my existence (p. 15).

At first, she says, “that acceptance was sad.” But soon she can tell herself: “you have moved on and stopped wanting what youth wants” (p. 24). And later, she sees real gains: “An important aspect of the ebbing of sex was that other things became more interesting. Sex obliterates the individuality of young women more often than it does that of young men, because so much more of a woman than of a man is used by sex …” (p. 39). Being childless and never married, Athill continues, she started to get glimpses of this freedom sooner than most “but not with the clarity I discovered once sex had fallen right away” (p. 40). There are also delightful gains in the possibility of friendship with men, without sexuality including, which she considers to be one of the privileges of old age.

These reflections make it clear that “ambiguity” while undermining the clear distinctions between the neoliberal “third” and “fourth” ages does not preclude the possibility of stages. Of course, there is movement through the life course and it has phenomenological significance (Andrews, 1999) but this is nuanced and complex and does not conform to the unidirectional “decline” narrative that configures the neoliberal life course. To put it phenomenologically, the past configures the present and as Merleau-Ponty suggests, provides it with a temporal atmosphere. So, while Athill certainly acknowledges that for her in many ways, “there’s no denying that moving through advanced old age is a downhill journey” (p. 179), yet that is not *all* it is. In the postscript she returns to the tree fern and describes it delightedly as bearing several fronds, which grow very slowly to begin with “but faster towards the end—so much faster that you can almost see it moving” (p. 183), which in itself can be read as an interesting simile for growth in her own life. In relation to the fern, she also expresses a fresh perspective in relation to her finitude, one that concurs with Beauvoir’s assertion of time’s value lying in its being spent and fulfilled: “I was right in thinking that I will never see it being a tree, but I underestimated the pleasure of watching it being a fern. It was worth buying” (p. 183).

In subsequent reflections on her life, Athill continues to find pleasure and exercise strengths in life as she grows more frail, is finally wheelchair-bound, and enters a residential home. Her experience of being in a wheelchair is as ambiguous as any other aspect of old age, certainly not the absolute misfortune we take for granted. At the age of 97 she says: “Nothing could be more deliciously luxurious than being pushed around a really thrilling and crowded exhibition in a wheelchair” (2015, p. 130), with crowds parting on either side of her to allow an intensely enjoyable and unimpeded view of her favorite pictures. Similarly, she extols the sensation of speed and ease in other settings such as “flashing” along in a wheelchair-bound queue in airports, which brings with it a feeling of euphoria doubtless invisible and inconceivable to younger observers (indeed she is particularly keen to communicate this unforeseen pleasure to old readers who may find the wheelchair symbolic of senescence and thus fear it). Finally, in talking about her decision to enter a “retirement home,” she talks about her situation in class and generational terms and in the context of never having made real decisions in her own life: “Few events in my life have been decided by me. How I was educated, where I have lived, why I am not married, how I have earned my living: all these crucial things happened to me rather than were made to happen by me.” By contrast, she enthuses, “Perhaps my decision to move into a home for old people is not quite the only one, but it is certainly the biggest” (2015, p. 99). Of course, Athill’s social and economic capital shore up this agency: her private residential home, The Mary Feilding Guild in Highgate, founded in the nineteenth century for unmarried professional women in their old age and badged on its contemporary website as providing “stimulating life in a convivial atmosphere of enquiring minds with some shared interests,” reminded Athill of her boarding school and clearly offered support for continued self-direction and self-expression within a supportive environment. As such, that this setting facilitates her continued growth and enables her to make choices that ensure she has a meaningful old age, is an experience that the older people living in less exceptional care homes are unlikely to share. However, both its presence now in her life and the fact that this sense of real choice comes so late in life suggest a vision of a deep old age more complicated than our hegemonic representations allow. Neither the third age nor the fourth, this is one of ambiguity, frailty combined with strength, agency not canceled out by enfeeblement, and care not negating independence.

### Florida Scott-Maxwell

A suffragist and feminist playwright born in 1883 in Florida, Scott-Maxwell’s most highly acclaimed work, *The Measure of My Days* (1968) was also, like Athill’s, her account of deep old age. Scott-Maxwell presents a sustained account of advanced old age as an increase in embodied energy, growth, and interpersonal connections *combined* with the constraints of weakness, illness, and isolation. Paradox consists in dwelling in a state of ambiguity containing all these possibilities. A week or so after an operation to remove her gall bladder she writes from hospital:

I got up threw back the curtains, opened all four windows—they would not open very wide—and expanded into the blue sky … … I was still eighty-two, they had done nothing about that, and I wanted to scale the sky … (p. 95).

She notes that there is not one homogeneous stage of old age but a variegated landscape with many such “stages”:

Here we come to a new place of which I knew nothing. We come to where age is boring, one’s interest in it by-passed; further on, go further on, one finds that one has arrived at a larger place still, the place of release (p. 140).

This unique configuring of the geography of deep old age, although written before the emergence of the third and fourth age binary, presents a richly alternative vision for movement through the latter part of the life course. So, her 80s represent a change from her 70s, but this is expressed in terms that defy categorization as either third or fourth age: “My seventies were interesting, and fairly serene, but my eighties are passionate. I grow more intense as I age” (p. 14). “Intensity” is a very interesting word used here to capture her vitality and “passion” for living, as she calls it, altogether foreign from biomedical terms. These surging ebbs and flows of energy encompass both frailty and strength, vigor and exhaustion, and are linked to an ambiguous dialectic between body and mind, flesh and will. So recovery from her gallbladder operation led to a fountaining of energy, and pain also spikes the body with vitality. She says, speaking collectively of older people, that despite their “drab” appearance “inside we flame with a wild life that is almost incommunicable” (p. 32). This wild life represents a certain increase in energy, not only from her 70s but from her middle age and youth: “perhaps the life I never lived, never guessed I had it in me to live” (p. 33). Paradoxically, this is, she thinks, precisely because she has withdrawn from social and practical involvement with the concerns of the outside world, disengaging from the usual struggles that accompany the roles and norms related to earlier life stages and decoupled from notions of success and failure. This then denotes the space we noted between world and response necessary for freedom according to Beauvoir; as Scott-Maxwell puts it: “It feels like the far side of precept and aim” (p. 33). For example, it means that, living on her own and unobserved by others, she can waltz on the shiny black and white linoleum floor in her kitchen waiting for the kettle to boil, free to express herself as she pleases where others “must vanish into their expected role” (p. 28). Old age is, for her, a process of integration and coherence *and* of being prepared to let it all go: “When at last age has assembled you together, will it not be easy to let it all go, lived, balanced, over?” (p. 42).

Throughout the pages of her memoir, Scott-Maxwell illuminates the situation of extreme old age: she expresses both a love of life and a willingness to die; a combined strength and frailty; a closeness and a distance from others; a life course without “use” to society which at the same time is replete with the possibility of growth and self-actualization. The existential reality of life now lies in the necessary interplay of contradictions: it is the emptiness of days that yet allow a “sense of vigour and spaciousness” (p. 141); it is the requirement to abandon “goals and efforts of a lifetime” (p. 119) that allows the discovery that growth and self-actualization occur within this pared-down simplified state from which all striving has been excised; it is good health that asserts itself in the midst of frailty and pain. She acknowledges the paradox, and reflects: “I know I am thinking two ways at once” (p. 119), but suggests that: “It is not being able to say conflicting things with one breath that is the sad division between human beings. As some dislike the paradoxical we forego the fun of admitting what we know …” (p. 142).

## Discussion and Concluding Thoughts

This paper proffers a framework through which to view the complexity of old age, one informed by the philosophy of ambiguity, which has not been discussed explicitly within the gerontological literature to date. Such a perspective enables a holistic way of encompassing the positive and negative aspects of age and of viewing them as inextricably intertwined. Through its conceptualization of oppression as denial, by the powerful, of this inherent ambiguity to others, together with the addition of new forms of constraining ambiguity, the framework also introduces a new perspective to the many-layered phenomenon of ageism. This perspective, applied to first-person accounts of life in deep old age, also offers a richer and more empowering view of choice and freedom than that provided by neoliberalism operating through discourses such as “successful aging.” Although memoirs of deep old age such as those by Athill and Scott-Maxwell remain rare, “ambiguity” as a principle can guide empirical data gathering in research into frailty, chronic illness, care homes, and other situations of deep old age as well as informing professional interactions. An approach firmly at home with uncertainty, of course it runs counter to the classificatory thrust of modernity and the latter’s desire, including through disciplinary fields and expertise, for crisp definitions and clear categorizations. Outside such categories it is possible to understand both the geography and the movement through the latter part of the life course in very different ways, and to make one’s own uniquely individual configurations. As Bauman notes: “ambivalence may be fought only with a naming that is yet more exact and classes that are yet more precisely defined” (Bauman, 1993, p. 3). He continues: “The world that thus falls apart into (a) plethora of problems is a manageable world” (p. 12). However, the philosophy of ambiguity reminds us, above all, that a manageable world is not a meaningful world, that one must and can make one’s own meaning including in deep age, and that it is by opening oneself up fully to the experience of deep old age, in all its risk and uncertainties, that the life course itself gains its richest significance.

## Data Availability

As this paper uses philosophical arguments, drawing on secondary texts only, the Transparency and Openness Promotion (TOP) Guidelines regarding replicability of data do not directly apply.
